# Deregulated mRNA and microRNA Expression Patterns in the Prefrontal Cortex of the BTBR Mouse Model of Autism

**DOI:** 10.1007/s12035-025-04900-x

**Published:** 2025-04-14

**Authors:** Catherine Mooney, Andrea Parlante, Giulia Canarutto, Andrea Grigoli, Maria Luisa Scattoni, Laura Ricceri, Eva Maria Jimenez-Mateos, Amaya Sanz-Rodriguez, Elena Clementi, Silvano Piazza, David C. Henshall, Giovanni Provenzano

**Affiliations:** 1 Department of Physiology & Medical Physics, RCSI University of Medicine and Health Sciences, Dublin, Ireland; 2 Computational Biology Unit, International Centre for Genetic Engineering and Biotechnology (ICGEB), Trieste, Italy; 3 Department of Cellular, Computational and Integrative Biology (CIBIO), University of Trento, 38123 Trento, Italy; 4 Research Coordination and Promotion Service, Istituto Superiore Di Sanità, Viale Regina Elena 299, 00161 Rome, Italy; 5 Centre for Behavioural Sciences and Mental Health, Istituto Superiore Di Sanità, Viale Regina Elena 299, 00161 Rome, Italy; 6 FutureNeuro Research Ireland Centre for Translational Brain Science, RCSI University of Medicine and Health Sciences, Dublin, Ireland; 7 Department of Life Sciences, University of Trieste, Trieste, Italy; 8 Present Address: School of Computer Science, University College Dublin, Dublin, Ireland; 9 Discipline of Physiology, School of Medicine, Trinity College Dublin, The University of Dublin, Dublin 2, Ireland

**Keywords:** Autism spectrum disorder, Prefrontal cortex, MRNA, MiRNA, Inflammatory pathways

## Abstract

Autism spectrum disorder (ASD) is a neurodevelopmental condition caused by both genetic and environmental factors. Since no single gene variant accounts for more than 1% of the cases, the converging actions of ASD-related genes and other factors, including microRNAs (miRNAs), may contribute to ASD pathogenesis. To date, few studies have simultaneously investigated the mRNA and miRNA profiles in an ASD-relevant model. The BTBR mouse strain displays a range of behaviors with ASD-like features but little is known about the protein-coding and noncoding gene expression landscape that may underlie the ASD-like phenotype. Here we performed parallel mRNA and miRNA profiling using the prefrontal cortex (PFC) of BTBR and C57BL/6 J (B6) mice. This identified 1063 differentially expressed genes and 48 differentially expressed miRNAs. Integration of mRNA and miRNA data identified a strong inverse relationship between upregulated (DEGs) and downregulated miRNAs, and vice versa. Pathway analysis, taking account of the inverse relationship between differentially expressed miRNAs and their target mRNAs highlighted significant shared enrichment in immune signaling, myelination, and neurodevelopmental processes. Notably, miRNA changes were predicted to affect synapse-related functions but we did not find enrichment of protein-coding genes linked to cellular components or biological processes related to synapses in the PFC of BTBR mice, indicating processes may evade miRNA control. In contrast, other miRNAs were predicted to have extensive relationships with DEGs suggesting their role as potential hub coordinators of gene expression. Profiling findings were confirmed via qRT-PCR for representative protein-coding transcripts and miRNAs. Our study underscores the complex interplay between gene expression and miRNA regulation within immune and inflammatory pathways in the BTBR model, offering insights into the neurodevelopmental mechanisms of ASD. These results support the value of the BTBR mouse model and identify strategies that could adjust molecular pathways for therapeutic applications in ASD research.

**Supplementary Information:**

The online version contains supplementary material available at 10.1007/s12035-025-04900-x.

## Introduction

Autism spectrum disorder (ASD) represents a heterogeneous group of neurodevelopmental conditions characterized by social interaction deficits, impaired communication, restricted interests, and stereotyped behaviors. The molecular mechanisms underlying ASD remain incompletely understood. Nevertheless, the heterogeneous clinical manifestations of ASD are thought to arise from a restricted set of pathophysiological processes [1–4]. This includes alterations in gene expression networks controlling synapse development, neuronal and glial cell activity, and immune function [1, 5–8].

MicroRNAs (miRNAs) are small noncoding RNAs (17–25 nucleotide long) that function as negative regulators of gene expression. The biogenesis of miRNA is well understood, and after processing from longer precursors, a miRNA is loaded into an argonaute protein, which directs binding to the 3′ untranslated region (UTR) of protein-coding transcripts, followed by translational repression and/or mRNA decay [9, 10]. There has been significant interest in the role of miRNAs in adjusting gene expression in neurodevelopmental disorders and in the pathogenesis of neuropsychiatric disorders [11], including ASD [12–14]. Notably, several studies have highlighted the crucial role of miRNAs in neural development [15]. In particular, miRNAs are essential for establishing and later maintaining cell diversity, morphology, and the structure of neurons. This includes a selection of miRNAs that regulate synaptic components within dendritic spines as well as an extensive range of pre-synaptic proteins and glial pathways that influence neurotransmission [16]. Altered levels of miRNAs have been detected in brain, blood, and saliva samples from subjects with ASD [17–21]. However, integrative approaches aiming to correlate large-scale mRNA and miRNA expression profiles in ASD are still lacking.

ASD mouse models represent a valid tool to study deregulated gene expression mechanisms at the network level. Adult BTBR T + Itpr3 tf/J (BTBR) mice are an inbred mouse strain that displays behavioral phenotypes relevant to the core symptoms of ASD, such as reduced social interactions, altered ultrasonic vocalizations, and repetitive stereotyped behaviors [22–26]. Beyond these behavioral traits, BTBR mice also show significant anatomical anomalies, such as agenesis of the corpus callosum and a dramatically reduced thickness and volume of the cortex, particularly in the prefrontal cortex (PFC) [27]. The PFC is crucial for the integration of complex brain processes such as social motivation and emotional state, as well as interpreting external cues in social interactions. Disruptions in PFC circuitry have been consistently linked to social behavior deficits typical of ASD [28], and studies in both human and animal models have underlined its significant role in social interactions [29]. Moreover, recent research has highlighted the PFC’s involvement in functional connectivity [30–32] and its impact on motor, interoceptive, and cognitive aspects of ASD [33]. The PFC displays molecular and functional impairments in both ASD subjects and mouse models. Indeed, gene expression alterations [34] and functional connectivity defects [35] have been detected in fronto-cortical areas of BTBR mice, in line with the altered molecular signatures [36] and functional connectivity deficits observed in the PFC of ASD subjects [37].

Detailed examinations of mRNA and miRNA profiles in ASD mouse models could provide critical insights into the genomic alterations that underlie the dysfunction of the PFC. Here, we performed parallel mRNA and miRNA profiling using the same RNA extracted from the PFC of BTBR mice. This enabled the integration of transcriptomic and miRNA data from the same brain region, thereby providing a comprehensive view of gene regulatory abnormalities in this mouse model of ASD. Our results indicate that the most significantly enriched pathways in the PFC of the BTBR mouse, across the differentially expressed mRNAs and miRNAs, were related to immune and inflammatory signaling, thus confirming the importance of these processes in ASD pathogenesis.

## Methods

### Animals

Experiments were conducted in conformity with the European Community Directive 2010/63/EU and approved by the Animal Welfare Committee of the National Institute of Health (Rome, Italy) and the Italian Ministry of Health. Animals were housed on a 12-h light/dark cycle with food and water available ad libitum. All efforts were made to minimize the number of animals used and animal suffering during the experiments. BTBR T + Itpr3 tf/J (BTBR) and C57Bl/6 J (B6) inbred mice were purchased from the Jackson Laboratory (Bar Harbour, ME, USA) and bred in the mouse vivarium of the Istituto Superiore di Sanità (Rome, Italy). A total of 18 adult (3–5 months old) male mice were used: 3 BTBR and 3 B6 mice for both mRNA microarray and miRNA OpenArray experiments and 6 mice per strain (BTBR, B6) for quantitative RT-PCR (qRT-PCR). Mice were weaned at postnatal day 21 and group-housed in standard mouse cages, with a maximum of 5 mice of the same sex and strain per cage. This housing arrangement was maintained until the point of euthanasia to minimize stress and potential confounding factors. All animals were euthanized at the same time across two different days to ensure consistency across experimental conditions and to minimize potential variability arising from circadian changes in gene expression.

### RNA Isolation

Total RNA was isolated from PFC samples using Qiazol (Qiagen, CA) and purified using the miRNeasy Kit (QIAGEN, CA) according to the manufacturer’s protocol. The quality of RNA was analyzed using microfluidic gel electrophoresis on RNA 6000 NanoChips, utilizing the Agilent 2100 Bioanalyzer. Only RNA with a high (> 9) RNA integrity number were selected and used for subsequent analysis. Each RNA sample was then divided into two aliquots that were used either for the gene expression microarray or miRNA profiling.

### Microarrays Analysis, Pathway/Phenotype Ontology, and Enrichment Analysis

Mouse gene expression arrays (Agilent 4 × 44 K slides) were hybridized and scanned with the Agilent microarray station, as previously described [38]. Images obtained from the microarray scanner were analyzed with Agilent Feature Extraction version 10.7.3.1. PFC gene expression dataset of BTBR and B6 mice have been deposited in the NCBI’s Gene Expression Omnibus (GEO) database (accession number GSE81502). Intensity values were processed with Agi4 × 44PreProcess using default parameters to remove low-quality probes. Differential gene expression analysis was done by using GeneSpring software (v.12.6), and raw signals were adjusted using quantile normalization without baseline transformation. Probes were filtered based on flag values. If the probes had a present or marginal value in at least one of the samples, the probes passed the filter. Data were further filtered on error (at least 2 out of 2 conditions have CV < 50.0%). The differential expression of probes was assessed using a moderated *t*-test with a *p*-value cutoff of 0.05. The *p*-values were adjusted for multiple testing using the Benjamini–Hochberg correction. A fold-change threshold of ≥ 1.5 was applied, consistent with previous studies that balance sensitivity and specificity to detect biologically meaningful changes in gene expression [39, 40].

### Pathway, Phenotype, and Enrichment Analysis of Differentially Expressed Genes (DEGs)

Pathway annotation analysis (KEGG pathway, reactome pathway, WikiPathways, and BioCarta) was carried out using DAVID 2021 (Dec. 2021 update) [41, 42]. Only pathway categories with a *p*-value < 0.05 were used for the figures.

As background for our functional analyses, we utilized the list of all genes identified as expressed in the PFC in this study. This list was compiled by filtering genes based on normalized expression values, excluding those with expression levels below the 10 th percentile, and ultimately included a total of 17,230 genes.

As a complementary approach, differentially expressed genes (DEGs) in BTBR PFC were analyzed for Mammalian Phenotype Ontology (MPO) using Enrichr (https://amp.pharm.mssm.edu/Enrichr/). Mammalian phenotypes predefined by Mouse Genome Informatics (MGI) were sorted by *z*-score considering only terms with an adjusted *p*-value less than 0.05. The direct enrichment analysis of different gene set categories on the DEGs and the predicted target genes of differentially expressed miRNAs (DEmiRNAs) from BTBR mice was performed by using the hypergeometric test present in R (*p*-value cutoff 0.05). Odds ratios were also calculated to quantify the strength of enrichment for each gene set. The background used to compute these enrichments was the same used for KEGG pathway analysis.

Details on the gene lists used for enrichment analyses, including their sources and corresponding genes, are provided in Supplementary Table 1. The selected gene lists include disease-related annotations for autism (e.g., genes from the SFARI database), as well as intellectual disabilities, and FMRP target genes. Cell-type markers for neurons, astrocytes, oligodendrocytes, and microglia were also considered. Further gene sets for enrichment analysis were obtained from a human transcriptome study on ASD, schizophrenia, and bipolar disorder [43], referred to as ASD_Gandal, SCZ_Gandal, and BD_Gandal. Two ASD-associated co-expression modules identified in transcriptomic studies of post-mortem cortical tissue from ASD patients [8] were also included in the analysis. These modules are asdM12, which is downregulated in ASD and enriched for genes involved in neuronal and synaptic functions, and asdM16, which is upregulated in ASD and enriched for genes involved in immune and inflammatory responses. Additionally, transcriptome and proteomic datasets from studies on BTBR mice [34, 38, 44–48] were used to complement the analysis. Additional gene enrichment analyses were performed using g:Profiler (with FDR as correction methods), Enrichr (for SynGO database), and Ingenuity Pathway Analysis (IPA). Terms with an adjusted *p*-value < 0.05 were reported.

### MiRNA Array Profiling Using OpenArray Platform

MiRNA profiling was performed using the TaqMan™ Rodent MicroRNA A Array v2.0 (Thermo Fisher Scientific; Catalog No. 4398967). RNA purified (100 ng) from the PFC of BTBR and B6 mice (*n* = 3 per group) was processed by reverse transcriptase and pre-amplification steps following the manufacturer’s protocol (Thermo Fisher Scientific). A 1:40 dilution of the pre-amplification reaction was mixed with TaqMan OpenArray Real-Time PCR Master mix (1:1). The mix was loaded onto the OpenArray rodent panel (750 mouse/rat miRNAs) using Open Array Accufill System and ran using a QuantStudio 12 K Flex PCR (Thermo Fisher Scientific), as described in previous studies in mice [49].

### MiRNA Expression Data Analysis

All analyses were performed in R Bioconductor [R Core Team, 2016; 50]. MiRNA expression datasets of BTBR and B6 mice have been deposited in NCBI’s GEO database (accession number GSE87601). The data was filtered as follows: if the cycle threshold (Ct) score for any miRNA in any sample was > 35, then the Ct score was set to 40 (i.e., it was considered “Undetermined”). If the Ct was “Undetermined” in 4 or more samples for any miRNA, then that miRNA was removed. The data was quantile-normalized and re-filtered by setting any Ct score that was > 30 to 40 (i.e., “Undetermined”). Again, if the Ct was “Undetermined” in 4 or more samples for any miRNA, then that miRNA was removed. Using these criteria, 306 miRNAs remained for the final data analysis. Differential expression analysis was performed by applying a Student’s *t*-test to the normalized Ct values between the two conditions, and the *p*-values were adjusted for multiple testing by controlling the false discovery rate (FDR) according to the method of Benjamini and Hochberg [51]. A miRNA was considered to be differentially expressed if the adjusted *p*-value was ≤ 0.05. The relative quantification (fold change, FC) was calculated as RQ = 2^−ΔΔCt^. To define differentially expressed miRNAs, we applied a threshold of FC > 2.0, consistent with previous studies that adopted similar criteria to capture biologically relevant changes in miRNA expression profiles [52].

### Enrichment and Pathway Analysis of DEmiRNAs

Experimentally validated or predicted with high confidence miRNA targets were provided by IPA and used for enrichment analysis. The direct enrichment analysis of different gene sets on predicted targets of up- and downregulated miRNAs was performed using the same approach and enrichment gene lists as for DEGs. DAVID 2021 [41, 42] was applied to conduct pathway enrichment analyses for the identified target genes. Only pathway categories with a *p*-value cutoff of 0.05 were used for the figures. IPA, Enrichr (for SynGO database) and g:Profiler enrichment analyses were conducted for DEmiRNAs and for predicted miRNA targets in the same way as DEGs, and only terms with an adjusted *p*-value < 0.05 were reported.

### DEmiRNA-DEG Relation Analysis

We analyzed the overlap between predicted DEmiRNA target genes and DEGs, performing hypergeometric tests with all expressed genes as the background to assess statistical significance. Next, the predicted interactions and regulatory networks between DEGs and DEmiRNAs were explored using IPA. DEGs and DEmiRNAs were imported into IPA, and relations between up and downregulated DEGs and DEmiRNAs were analyzed with two approaches, identifying the shortest path between nodes and then the shortest path plus one extra intervening node. The chi-square statistical test was subsequently used to evaluate the relationships between up- and downregulated elements.

### Quantitative RT-PCR (qRT-PCR)

For validation of microarray and OpenArray-based screening, PFC tissues from additional B6 and BTBR mice were dissected and frozen in dry ice. All samples were stored at − 80 °C until use. Total RNAs were extracted by Trizol reagent (Invitrogen), treated with DNAse, purified by the RNAeasy Kit (Qiagen), and pooled (*n* = 6 per genotype). For gene expression quantification, cDNA was synthesized from pooled RNAs by SuperScript VILO cDNA Synthesis Kit (Invitrogen) according to the manufacturer’s instructions. qRT-PCR was performed in a C1000 Thermal Cycler (Bio-Rad) with real-time detection of fluorescence, using the KAPA SYBR FAST Master Mix reagent (KAPA Biosystems). Mouse mitochondrial ribosomal protein L41 (mRPL41) was used as a standard for quantification. Primer sequences (Eurofins Genomics) are reported in Supplementary Table 2. Ratios of comparative concentrations of each mRNA with respect to L41 mRNA were then calculated and plotted as the average of three independent reactions (technical replicates) obtained from each RNA. For miRNA expression, reverse transcription for individual qPCR was carried out using 500 ng of total RNA and the TaqMan® MicroRNA Reverse Transcription Kit (Thermo Fisher Scientific). RT specific primers for miR-381, miR-27b, let-7b, miR-146a, and RNU19 (assay ID: 000571, 000409, 002619, 000468, 001003; Thermo Fisher Scientific) were used for miRNA reverse transcription. Individual qPCRs were carried out on the CFX96 Touch (Bio-Rad). RNU19 was used as a housekeeping for normalizing miRNA expression as previously reported [53, 54]. A relative fold change in expression of the target gene transcript was determined using the comparative cycle threshold method (2^−^ΔΔCT), as described previously [55]. Expression analyses were performed using the CFX3 Manager (Bio-Rad) software [56]. Statistical analysis of qRT-PCR was performed with Prism 6 (GraphPad) software. Values were expressed as mean ± SEM, and quantitative gene expression differences between BTBR and B6 controls were assessed by Student’s *t*-test, with the level of statistical significance set at *p* < 0.05.

## Results

### mRNA Profiling of the PFC in BTBR Mice

**Fig. 1:**
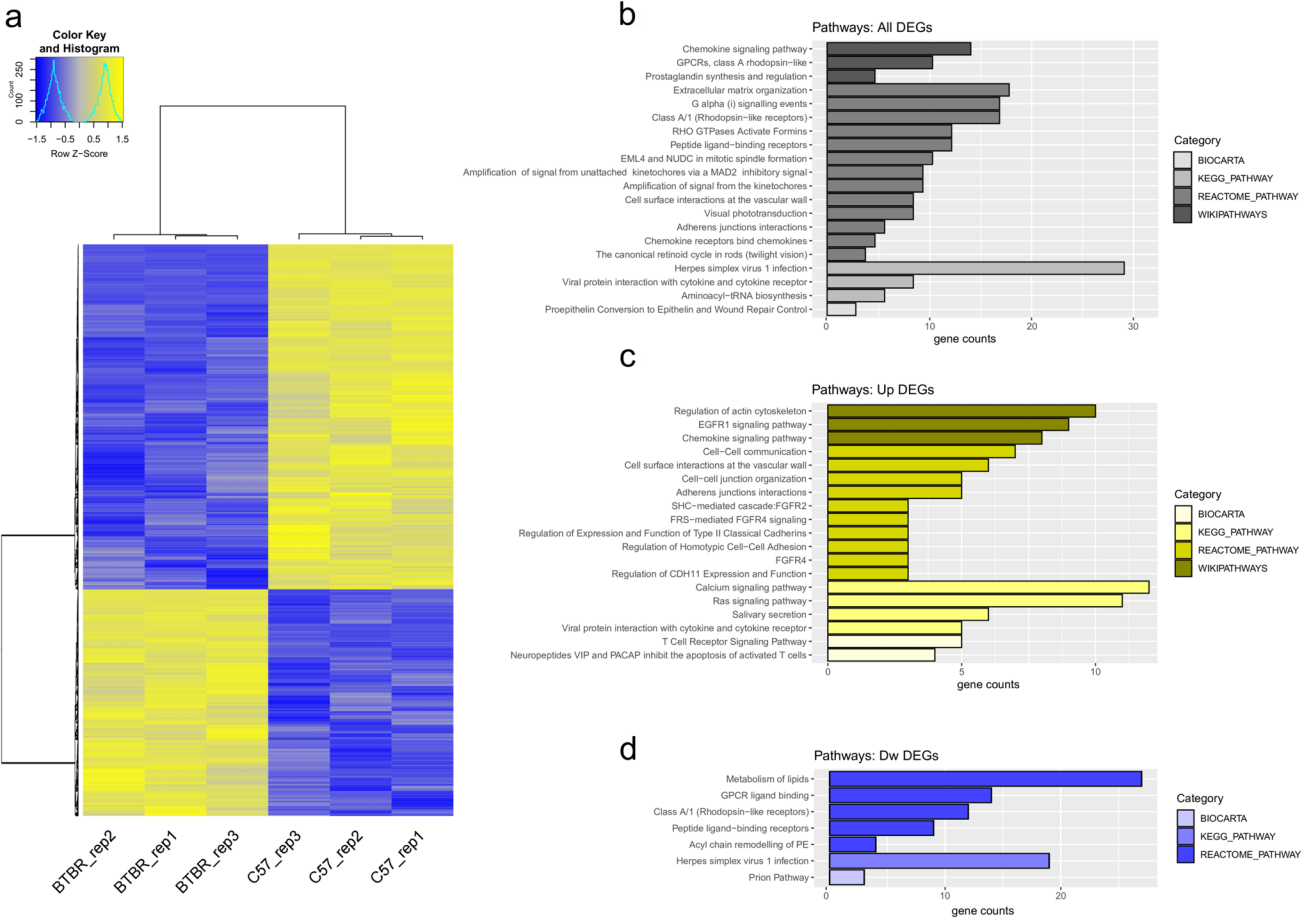
Hierarchical clustering representation and overrepresented pathway categories in the PFC of BTBR mice.** a** Heatmap showing hierarchical clustering of 1063 differentially expressed genes in the prefrontal cortex between BTBR and B6 control mice. A color-coded scale was used to show gene expression differences in logarithmic fold change units between the groups (blue represents the lowest expression; yellow represents the highest expression)(b-d) Overrepresented pathway categories for differentially expressed genes in the BTBR PFC: all DEGs (**b**), up-regulated (Up) DEGs (**c**), and down-regulated (Dw) DEGs (**d**). DEGs were analysed for enrichment in pathway categories using DAVID, with a p-value < 0.05. For each term, the number of genes is indicated by the length of horizontal bars (gene counts).

To identify genes and pathways differentially expressed in the PFC brain tissue of BTBR mice, we performed comprehensive transcriptome profiling. PFC samples from BTBR and B6 control adult mice were assessed for differential gene expression by microarray and bioinformatic analysis. A total of 1063 genes were differentially expressed in the PFC of BTBR mice, as compared to B6 controls (Fig. 1a). Among these, 410 and 653 were up- and downregulated, respectively. Supplementary Table 3 shows the entire list of DEGs in the BTBR PFC, with fold change and adjusted *p*-values.

### Functional Analysis of Differentially Expressed Genes

Next, we explored the pathway/phenotype ontologies for the DEGs in the PFC of BTBR mice through DAVID (The Database for Annotation, Visualization and Integrated Discovery), using KEGG (Kyoto Encyclopedia of Genes and Genomes), Reactome, WikiPathways, and BioCarta as databases. The background list used for the enrichment analysis is reported in Supplementary Table 4, while the complete results of DAVID DEG analysis (for all, up-, and downregulated genes) are reported in Supplementary Table 5. The pathways that were most significantly enriched across all DEGs of BTBR mice include chemokine signaling, extracellular matrix organization, and adherens junction interactions (Fig. 1b). A significant over-representation of pathways related to the regulation of actin cytoskeleton, cell–cell adhesion and communication, expression/function of type II classical cadherins, calcium-, chemokine-, and T cell receptor-signaling, and inhibition of activated T cell apoptosis was found among BTBR upregulated DEGs with the lowest *p*-values (Fig. 1c; Supplementary Table 5). Conversely, metabolism of lipids, GPCR ligand binding, and prion pathway were detected among downregulated DEGs in BTBR mice (Fig. 1d; Supplementary Table 5).

**Fig. 2:**
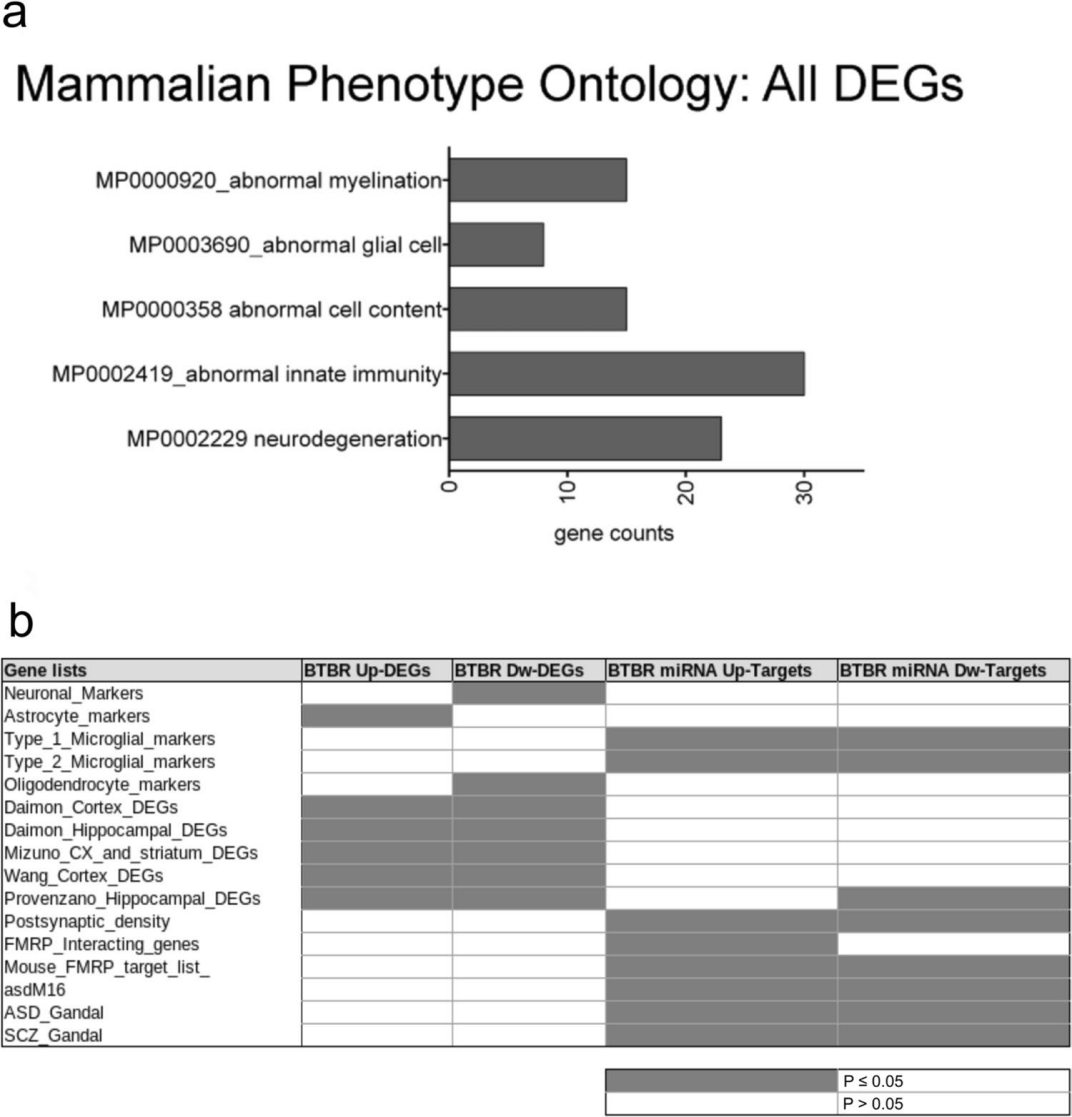
Mouse phenotype categories and enrichment analysis on BTBR PFC DEGs. (**a**) PFC BTBR DEGs were analyzed for enrichment in mouse phenotypes using Enrichr with a *p*-value cutoff of 0.05. The top five significant phenotype categories found in the BTBR DEGs are arranged from the most to the least significant. The number of genes in the category is indicated by the length of the horizontal bars (gene counts). (**b**) Up- and downregulated DEGs and DEmiRNAs were subjected to enrichment analysis for cell-type specific markers, neurodevelopmental and neurodegenerative disease terms, and transcriptomic datasets derived from patients or mouse models of neurodevelopmental disorders. Enrichment significance is represented by color, with white for *p* > 0.05 and gray for *p* < 0.05

We then tested BTBR PFC DEGs for the enrichment of mouse phenotype terms (Mouse Genome Informatics Mammalian Phenotype Level 4) using Enrichr (Fig. 2a). We found significant enrichment for terms related to abnormal myelination, glial cells, innate immunity, and neurodegeneration.

To complement our functional analysis and provide further insight into the interpretation of the transcriptome data, we tested whether the BTBR DEGs were specifically enriched for cell-type specific markers, genes implicated in neurodevelopmental diseases, and those previously found to be dysregulated in transcriptomic studies of autism, bipolar disorder, and schizophrenia, as well as in other transcriptomic studies conducted on BTBR mice across different brain regions (Supplementary Table6). The DEGs from the PFC of BTBR mice showed significant enrichment in cell-type-specific markers, with upregulated genes predominantly associated with astrocyte markers and downregulated genes enriched for oligodendrocyte and neuronal markers (Fig. 2b). Both up- and downregulated DEGs showed significant overlap with gene lists from previous transcriptomic studies on BTBR mice, particularly those focusing on the hippocampus and cortex [38, 44–46, 48]. No significant enrichment was observed for autism risk genes from the SFARI database or for genes associated with other neuropsychiatric disorders such as intellectual disability, bipolar disorder, and schizophrenia (Supplementary Table 6).

In addition, we performed ontology enrichment analysis using g:Profiler to further explore ontologies deregulated in the PFC of BTBR mice. This analysis (Supplementary Table 7, Supplementary Fig. 1) revealed several significant terms (adjusted *p*-value < 0.05), with upregulated DEGs strongly associated with actin cytoskeleton regulation, neuron projection development, and cell adhesion and downregulated DEGs linked to neurodevelopmental processes such as axon ensheathment, regulation of myelination, and oligodendrocyte differentiation, as well as pathways related to lipid metabolism and oxidative stress. These results are consistent with the other enrichment analyses, providing additional insights into the cellular and molecular pathways contributing to PFC dysfunction. A targeted analysis of genes with synaptic functions using SynGO [57], a knowledge base and platform for evidence-based synaptic annotations, revealed that the DEGs did not show significant enrichment in cellular components or biological processes related to synapses.

### miRNA Profiling in the PFC of BTBR Mice

**Fig. 3:**
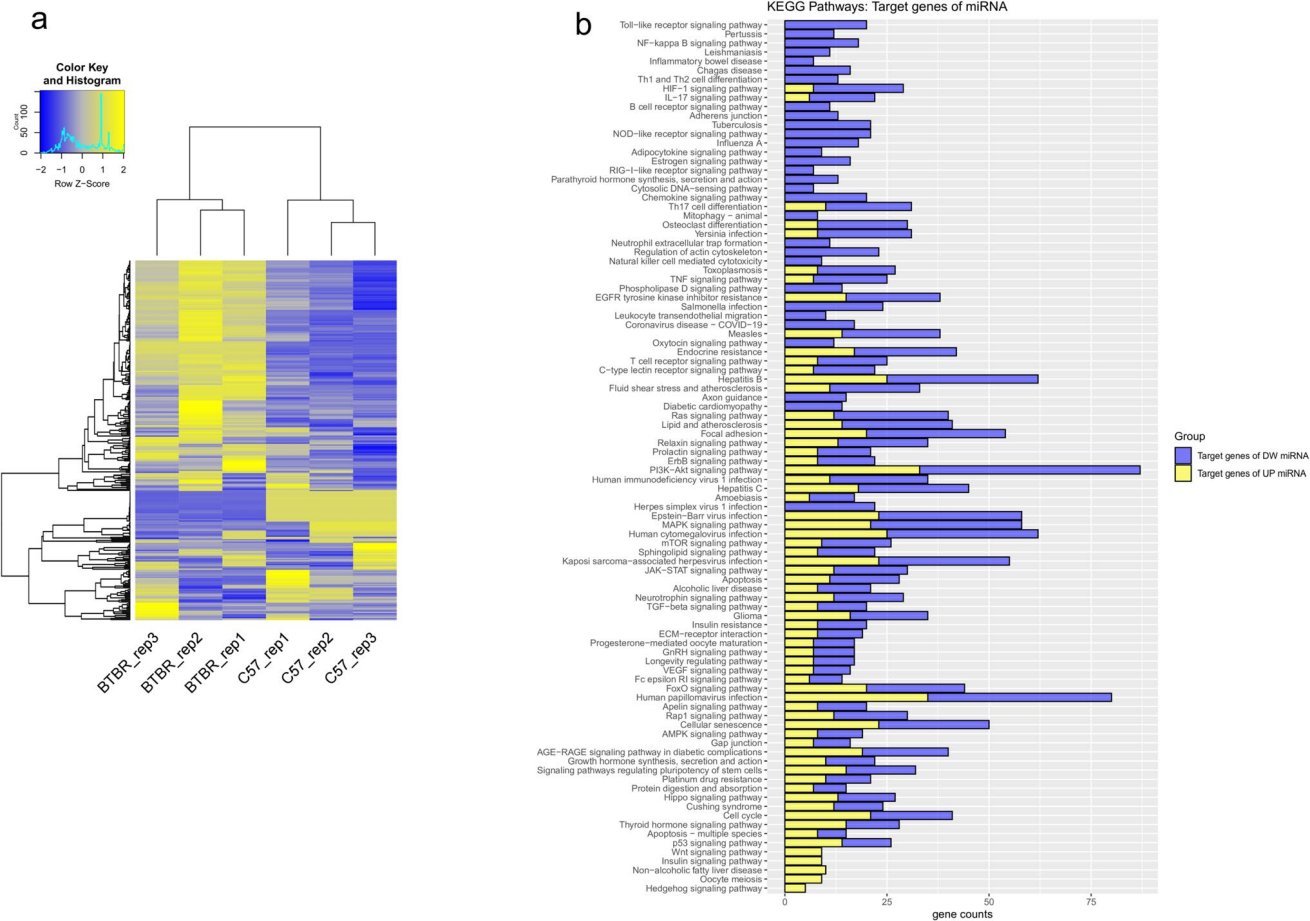
Heat map of DEmiRNAs and KEGG pathway enrichment analysis of predicted miRNA targets. (**a**) Heat map of the 48 differentially expressed miRNAs, with unsupervised hierarchical clustering by miRNAs, in the prefrontal cortex between BTBR and B6 mice. The dendrogram was constructed using the complete linkage method with the Euclidean distance measure. The analysed samples are shown in columns, and the DEmiRNAs are presented in rows. Yellow represents increased expression and blue represents reduced expression. (**b**) KEGG pathway analysis of the predicted target genes for up- and down-regulated DEmiRNAs in the PFC between the BTBR and B6 control mice. DEmiRNA target genes were analysed for enrichment in KEGG pathways using DAVID with a p-value cutoff of 0.05. The x-axis shows the counts of DEmiRNA target genes enriched in KEGG pathways, and the y-axis shows the KEGG pathways. Yellow bars represent the target genes of upregulated miRNAs, while blue those of down-regulated miRNAs. KEGG pathways are ranked from top to bottom based on the difference between down- and up-regulated miRNA gene targets in the ratio of gene counts to the number of genes in the pathway (Population Hits)

We next profiled miRNAs by high-throughput OpenArray platform, analyzing the same RNA extracted from PFC tissue samples of BTBR and B6 mice that were used for mRNA microarray analysis. We identified a total of 48 deregulated miRNAs in the PFC of BTBR mice compared to their B6 controls. Among them, 25 miRNAs were significantly upregulated, and 23 were significantly downregulated. Expression values for all 48 significantly deregulated miRNAs were clustered for each individual mouse, and a heat map was derived using unsupervised hierarchical clustering (Fig. 3a). The full list of statistically significant DEmiRNAs with fold change and adjusted *p*-values is provided in Supplementary Table 8.

Among the DEmiRNA in the PFC of BTBR mice, we identified multiple miRNAs previously associated with experimental and human ASD. This included miR-106b, miR-486, miR-27b, miR-381, miR-146a, miR-23a, miR-328, let-7b, and miR-15b [58, 59]. Previous studies have demonstrated that miR-23a, miR-328, and miR-381 regulate ASD-related genes such as NRXN1 and SHANK3 [58]. We also observed dysregulation of miR-592 and miR-93 which were implicated in ASD-associated pathways, with miR-592 contributing to inflammatory signaling and miR-93 playing a role in neuronal plasticity [60, 61].

### Functional Analysis of Differentially Expressed miRNAs

To explore the functional effects of the DEmiRNAs in the PFC of BTBR mice, we integrated miRNA target prediction and pathway enrichment analysis. The predicted target genes (experimentally validated or predicted with high confidence) for each of the 48 deregulated miRNAs were generated with IPA and analyzed for enriched terms associated with cell-type-specific markers and genes involved in neurodevelopmental diseases, as well as pathways and gene ontologies retrieved by DAVID, g:Profiler and SynGO. As an initial analysis, we built a comprehensive list of all predicted target genes of DEmiRNAs, reported in Supplementary Table 9, and we assessed them for enrichment in gene sets, ontologies, and pathways. The predicted target genes of both up- and downregulated DEmiRNAs showed significant enrichment across several gene sets associated with categories relevant to the autistic phenotype (Fig. 2b). Specifically, we observed enrichment for genes associated with postsynaptic density, microglial markers, and asdM16 a co-expression module dysregulated in autism brains and specifically enriched in genes predominantly expressed by astrocytes and microglia [8]. Furthermore, we observed a significant overlap with human transcriptomic studies, specifically with gene lists associated with ASD and schizophrenia [43]. Interestingly, the predicted targets of the upregulated DEmiRNAs significantly overlapped with FMRP-target genes, suggesting a potential convergence on synaptic regulatory mechanisms that are known to be altered in fragile X syndrome. Notably, the targets of downregulated miRNAs were significantly enriched for genes previously found to be dysregulated in the hippocampus of BTBR mice [38]. Similar to the analysis performed on DEGs, the predicted targets of the DEmiRNAs did not show significant enrichment for autism risk genes from the SFARI repository or for genes associated with neuropsychiatric disorders such as intellectual disability, bipolar disorder, and schizophrenia. Results of the enrichment analysis, including overlap *p*-values and odds ratios, are reported in Supplementary Table 6.

KEGG pathways were analyzed for up- and downregulated miRNAs (Fig. 3b) and considered significant when their *p*-values were < 0.05. The Wnt and insulin signaling pathways were shown to be among the most associated with upregulated miRNA target genes. Conversely, the top pathways for the downregulated miRNA were related mainly to T and B cell differentiation and signaling, chemokine signaling pathway, regulation of actin cytoskeleton, adherens junction, and axon guidance. Of note, a number of KEGG pathways were significantly enriched for both up- and downregulated miRNA target genes, including focal adhesion, MAPK, and PI3 K-Akt signaling pathways. Complete results of DAVID pathway analysis on target genes of DEmiRNAs are reported in Supplementary Table 10.

**Fig. 4:**
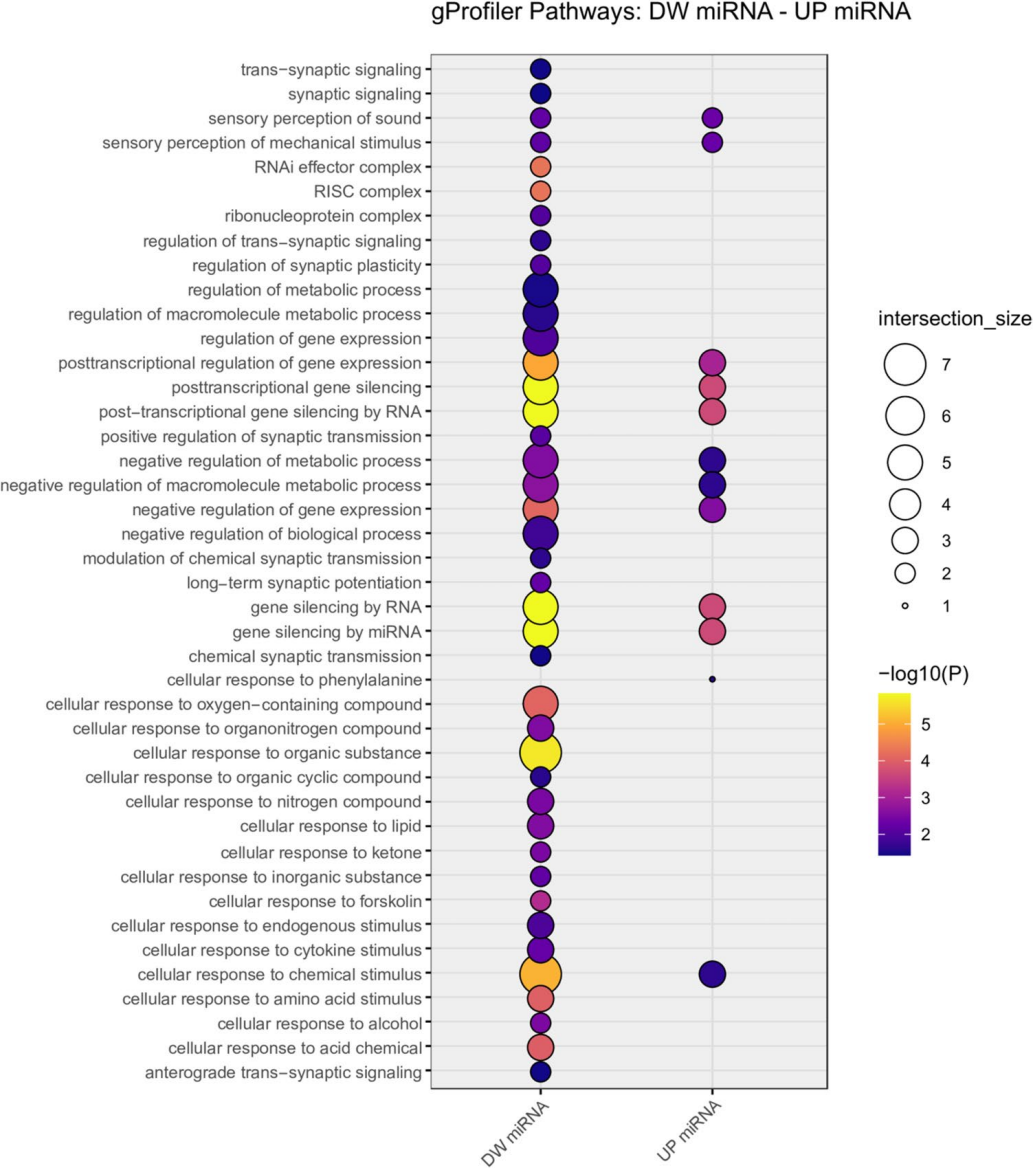
Balloon plot representing the most significant results of g:Profiler analysis onDEmiRNAs. The *x*-axis distinguishes between down- and upregulated miRNAs, while the *y*-axis lists significant biological terms. The size of the balloons is proportional to the number of miRNAs involved in each biological pathway, and the color gradient and intensity reflect the significance of the enrichment, represented by the negative logarithm of the corrected p-value

We also performed a functional analysis on DEmiRNAs using g:Profiler, as reported in Supplementary Table 11. In this functional gene ontology analysis, downregulated DEmiRNAs were shown to be enriched with several terms related to trans-synaptic signaling, long-term synaptic potentiation, regulation of synaptic plasticity, and synaptic transmission (Fig. 4). Conversely, upregulated DEmiRNAs were enriched with terms related to metabolic processes and did not show any synapse-related term (Fig. 4). Moreover, SynGO analysis confirmed a significant enrichment (adjusted *p*-value ≤ 0.05) in synapse-related cellular components and biological processes for the predicted targets of DEmiRNAs (Supplementary Table 12). Most of the enriched terms were found among the targets of downregulated DEmiRNAs, which showed enrichment in postsynaptic specialization and in the regulation of postsynaptic and presynaptic assembly.

### Shared Functional Features Between mRNA and miRNA Enrichment Analyses

Since we hypothesized there may be an inverse correlation between the expression of miRNAs and their corresponding mRNAs [62], we investigated a potential functional relationship between mRNAs and miRNAs, observing which pathways were shared between the enrichment results of upregulated DEGs and gene targets of downregulated DEmiRNAs and between the enrichment results of downregulated DEGs and gene targets of upregulated DEmiRNAs.

**Fig. 5:**
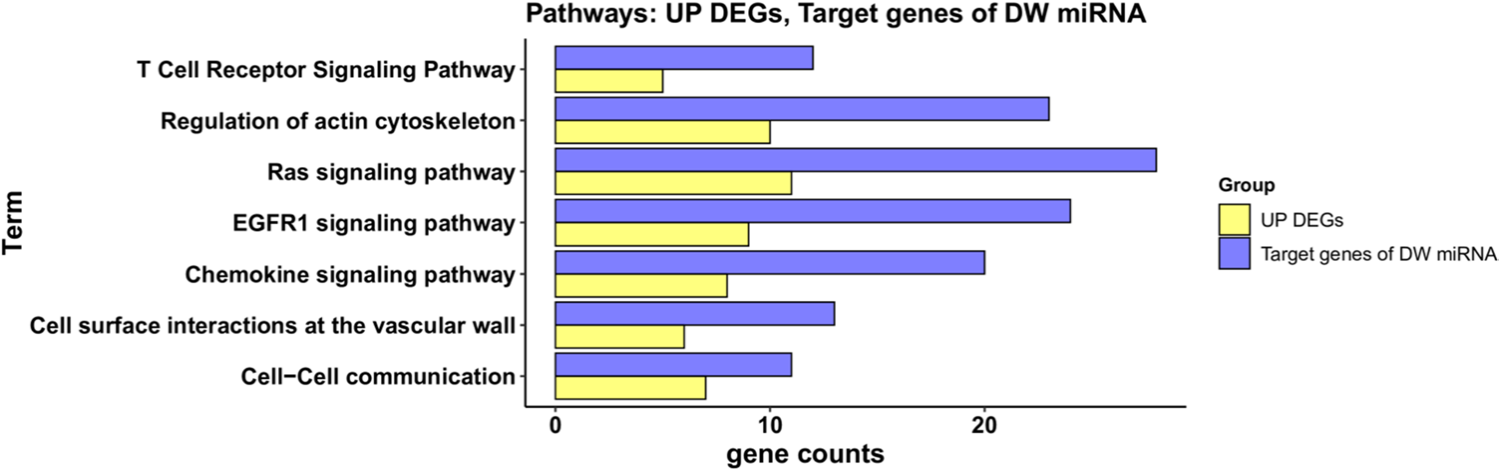
DAVID pathway analysis for upregulated DEGs and target genes of downregulated DEmiRNAs. Only pathways with a significant *p*-value (< 0.05) that were shared between the two groups were considered. For each pathway, the number of genes is indicated by the length of horizontal bars (gene counts). Yellow bars represent upregulated DEGs, while blue bars indicate target genes of downregulated DEmiRNAs

From DAVID pathway analysis, we found a convergence for upregulated DEGs and target genes of downregulated DEmiRNAs in the T cell receptor signaling pathway, chemokine signaling pathway, regulation of actin cytoskeleton, Ras signaling pathway, EGFR1 signaling pathway, cell surface interactions at the vascular wall, and cell–cell communication (Fig. 5). On the other hand, no significant pathways were found to be shared between downregulated DEGs and target genes of upregulated DEmiRNAs using DAVID.

**Fig. 6:**
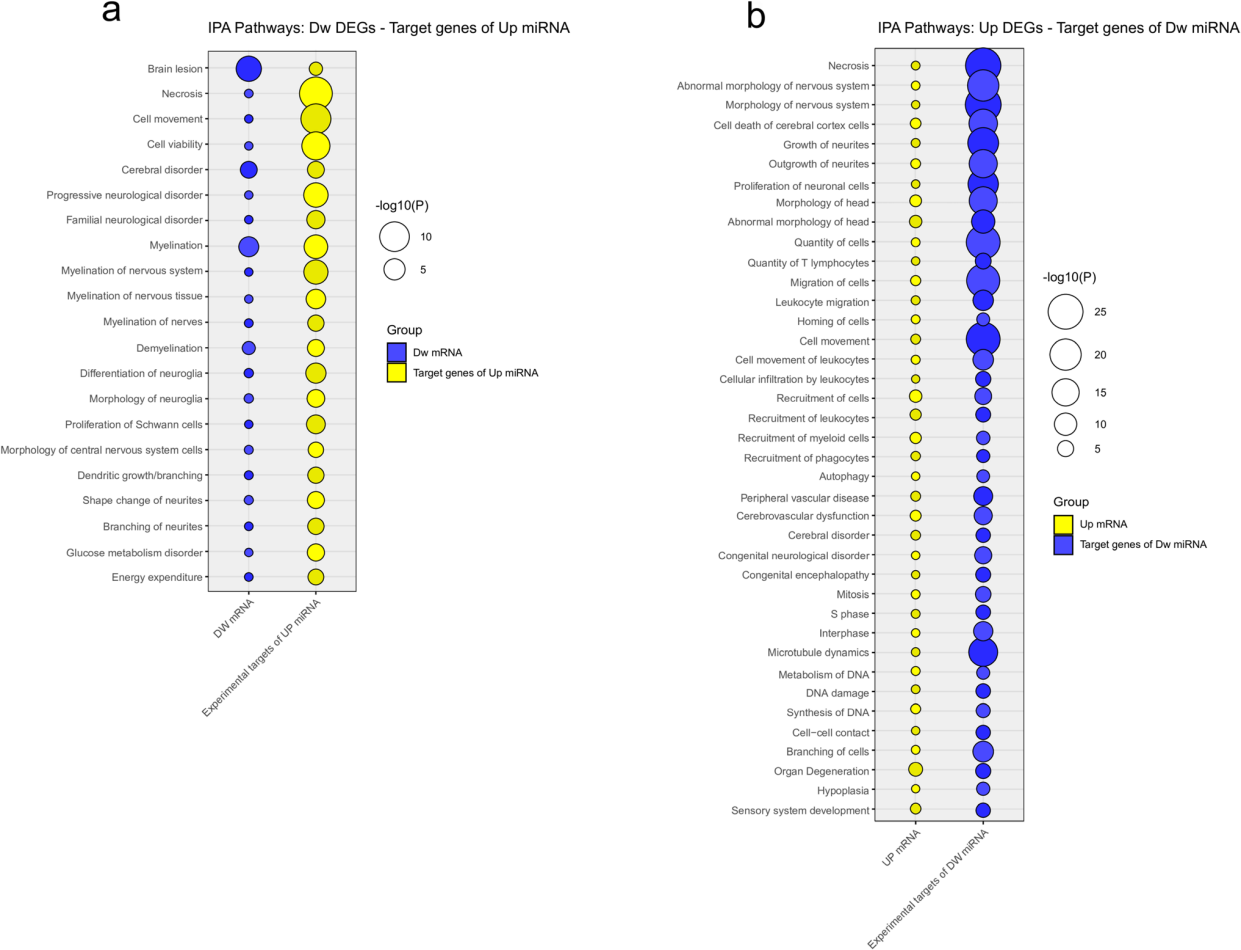
IPA functional analysis for (**a**) downregulated DEGs and target genes of upregulated DEmiRNAs and (**b**) upregulated DEGs and target genes of downregulated DEmiRNAs. Only pathways with a significant adjusted *p*-value (< 0.05) that were shared between the two groups were considered. Yellow balloons indicate upregulation, while blue balloons indicate downregulation. For each function, reported on the *x*-axis, the balloon size is proportional to the negative base 10 logarithm of the adjusted *p*-value (-log10(P))

The IPA functional enrichment analysis showed shared features between downregulated DEGs and target genes of upregulated DEmiRNAs mainly regarding myelination, differentiation, and morphology of neuroglia, dendritic branching, and energy expenditure (Fig. 6a). Conversely, abnormalities in the quantity of T cells, migration and recruitment of leucocytes, sensory system development, morphology of the nervous system, autophagy, cell cycle, and microtubule dynamics were shared among upregulated DEGs and target genes of downregulated DEmiRNAs (Fig. 6b). Supplementary Table 13 reports all functions retrieved from IPA analysis for up- and downregulated DEGs, DEmiRNAs, and target genes of DEmiRNAs, where values represent the negative base 10 logarithms of the adjusted *p*-value.

**Fig. 7:**
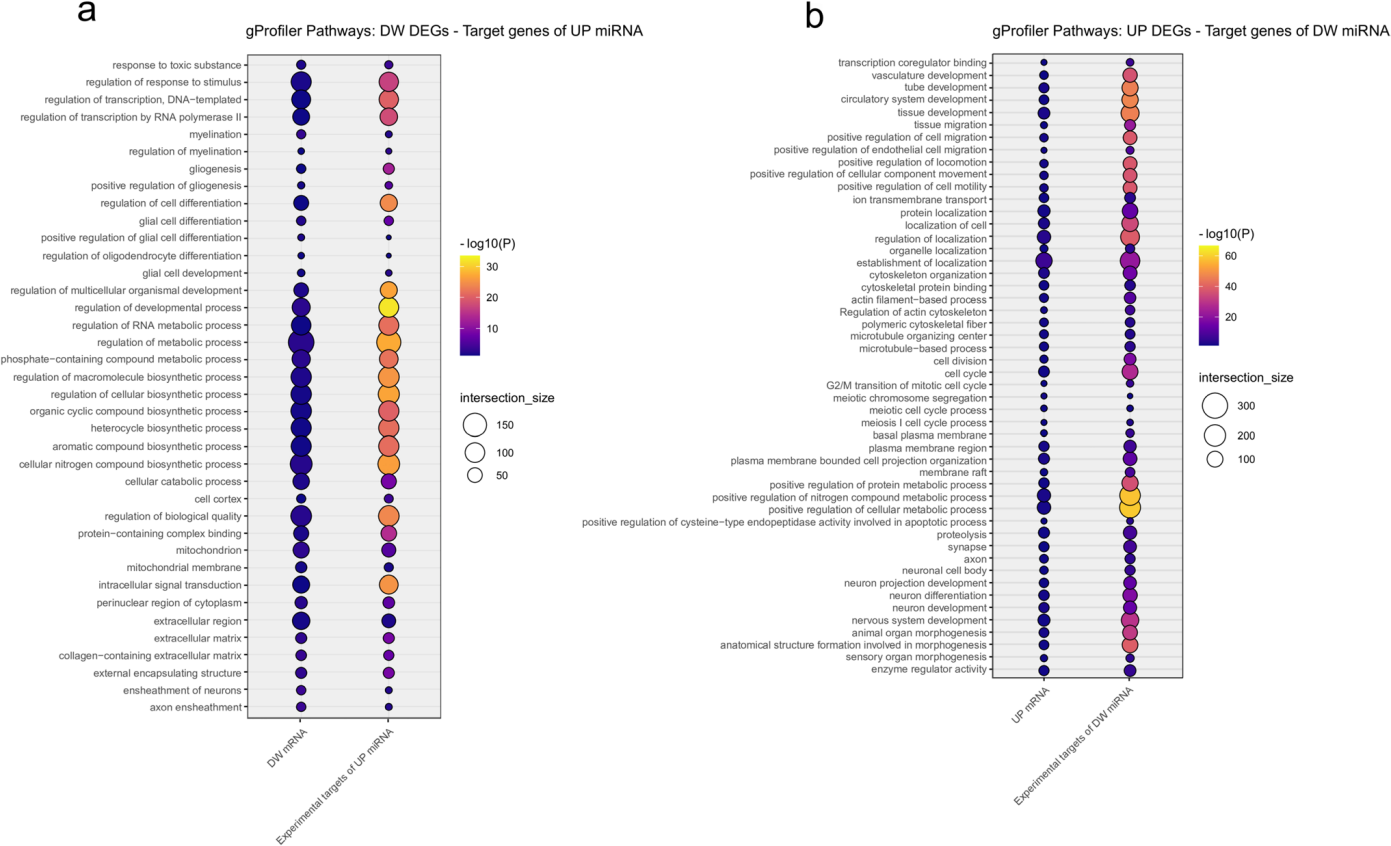
g:Profiler functional analysis for (**a**) downregulated DEGs and target genes of upregulated DEmiRNAs and (**b**) upregulated DEGs and target genes of downregulated DEmiRNAs. Only pathways shared between the two groups and with a significant adjusted *p*-value (< 0.05) were considered. For each biological term, reported on the *y*-axis, the size of the balloons is proportional to the number of genes involved in the biological pathway, while the color gradient and intensity reflect the significance of the enrichment, represented by the negative logarithm of the corrected *p*-value

Enrichment analysis using g:Profiler on DEGs and target genes of DEmiRNAs was performed to further investigate the functional relationships between mRNAs and miRNAs. The results (Supplementary Table 7, Fig. 7a, b) revealed several significant terms (adjusted *p*-value < 0.05), many of which showed significant overlap with terms identified through IPA analysis. Specifically, we identified shared dysregulations between downregulated DEGs and target genes of upregulated DEmiRNAs in processes related to myelination, genesis and differentiation of glial cells (in particular oligodendrocytes), mitochondria, and extracellular matrix (Fig. 7a). On the other hand, terms related to cell localization, movement and migration, cytoskeleton and microtubule organization, cell cycle, vasculature development, and neuronal differentiation and development were found to be shared between upregulated DEGs and target genes of downregulated DEmiRNAs (Fig. 7b).

### DEmiRNA-DEG Relation Analysis

Since many miRNAs might act as repressors of target mRNAs by promoting their destabilization and degradation [63, 64], we performed a miRNA–mRNA intersection analysis to investigate the potential functional relationships between upregulated DEmiRNAs and downregulated DEGs, and vice versa. To test this, we assessed the overlap between the predicted target genes of DEmiRNAs, retrieved from IPA, and our DEG profiles, matching them to inverse correlation.

The analysis revealed 13 overlapping genes in the downregulated DEGs (DEGs_DW) vs. miRNA upregulated (miRNA_UP) target correlation and 15 in the upregulated DEGs (DEGs_UP) vs. miRNA downregulated (miRNA_DW) target correlation (Supplementary Table 9). Although none of these overlaps reached statistical significance, several genes within the DEGs_UP_vs_miRNA_DW_target correlation were associated with inflammation, including *Cxcr4*, *Ccnd1*, *Cd47*, *Ets1*, *Grn*, *Nfkb1*, and *Ptgs2* (Supplementary Table 9).

These findings highlight the need for a more integrative approach to investigate the potential interactions between DEGs and DEmiRNAs. To achieve this, we utilized IPA to integrate expression data from DEGs and target genes of DEmiRNAs with predicted interactions, seeking to elucidate their complex interplay. Our analysis considered all potential interactions across four groups (i.e., DEGs up, DEGs down, DEmiRNAs up, DEmiRNAs down). Results are summarized in Supplementary Table 14, which includes both the shortest path and shortest path + 1 analyses. Unique relationships identified by IPA were used to calculate chi-square statistics, providing a quantitative measure of the association between these groups.

**Fig. 8:**
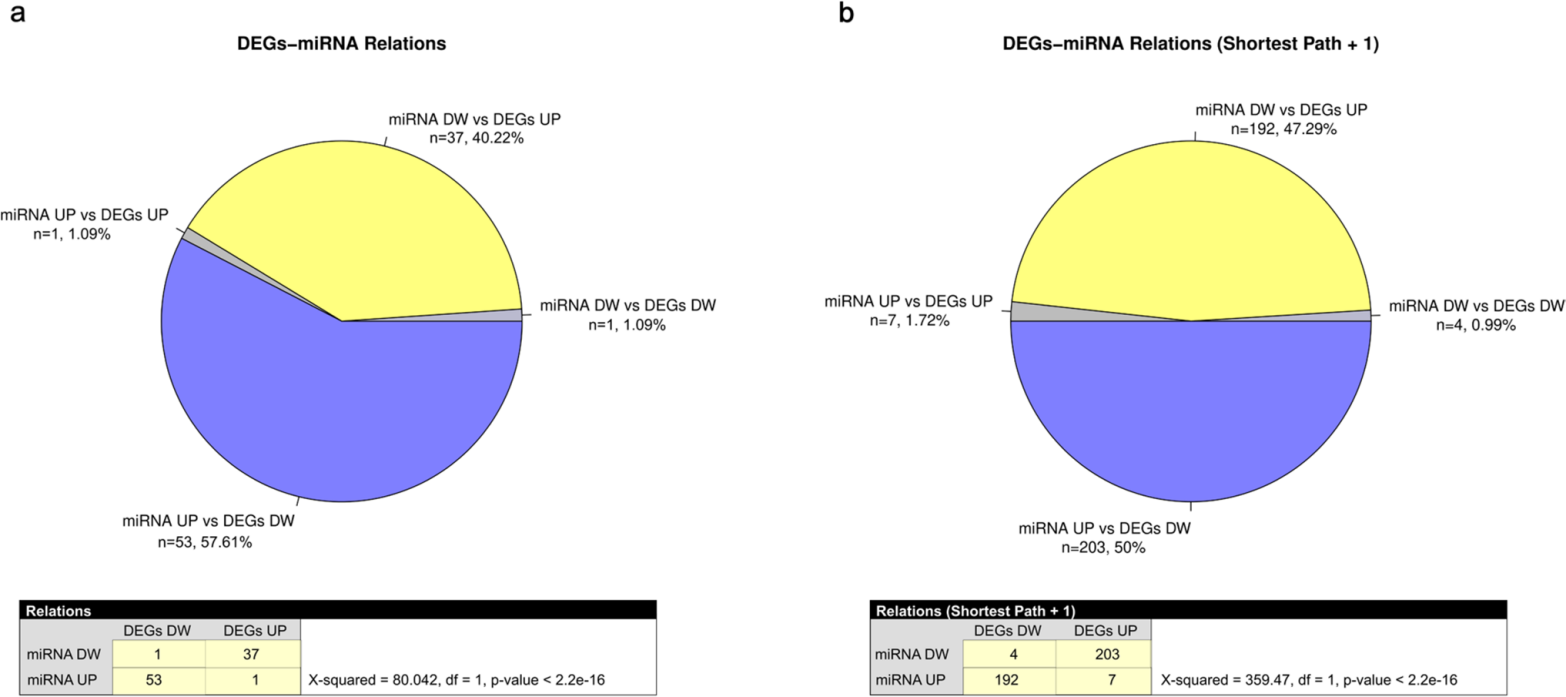
DEmiRNA-DEG relation analysis. Pie charts illustrate the percentage and size of unique relationship retrieved with IPA between different combinations of up- and downregulated DEGs and DEmiRNAs. (**a**) The left chart shows results using the shortest path between nodes. (**b**) The right chart includes the shortest path plus one additional intervening node. The tables below the pie charts summarize the size of the relationships and the chi-square significance values

Significant chi-square values were observed for both the shortest path and shortest path + 1 analyses (*X*^2^ = 80.042, df = 1, *p*-value < 2.2e- 16, and *X*^2^ = 359.47, df = 1, *p*-value < 2.2e- 16, respectively), as shown in Supplementary Table 14 and Fig. 8. These results highlight a strong correlation between upregulated DEGs and downregulated DEmiRNAs, as well as between downregulated DEGs and upregulated DEmiRNAs. This pattern underscores regulatory dynamics, emphasizing the interplay between mRNA and miRNA expression in the PFC of BTBR mice. The shortest path analysis showed that miRNA upregulation was predominantly associated with DEG downregulation (57.61%, *n* = 53), while miRNA downregulation was linked to DEG upregulation (40.22%, *n* = 37). Incorporating one additional intervening node in the shortest path + 1 analysis revealed a broader interaction network. Upregulated miRNAs were linked to downregulated DEGs in 50% of interactions (*n* = 203), while downregulated miRNAs were linked to upregulated DEGs in 47.29% of interactions (*n* = 192). These findings reflect an increased number of interactions while maintaining the overall inverse regulatory trend between miRNAs and DEGs.

Of particular interest, miRNAs such as let-7b, miR-143, and miR-34b emerged as prominent regulatory hubs due to their recurrent predicted interactions within the dataset (Supplementary Table 14). These miRNAs exhibited extensive connections with interactors such as *Myc*, *Tp53*, *E2f1*, *Npm1*, *Bmi1*, *E2f2*, *Rela*, *Relb*, *Pparg*, *Bcl6*, *Runx1*, *Spi1*, *Ar*, *Stat1*, *Nfkb1*, and *Smad3*, which are critical regulators of neurogenesis, myelination, and immune responses. Notably, upregulated miRNA let- 7b showed the highest number of interactions with downregulated DEGs, suggesting its potential role in the pathogenesis and molecular mechanisms underlying ASD-like behaviors observed in BTBR mice.

### qRT-PCR Validation of Differentially Expressed mRNAs and miRNAs

**Fig. 9:**
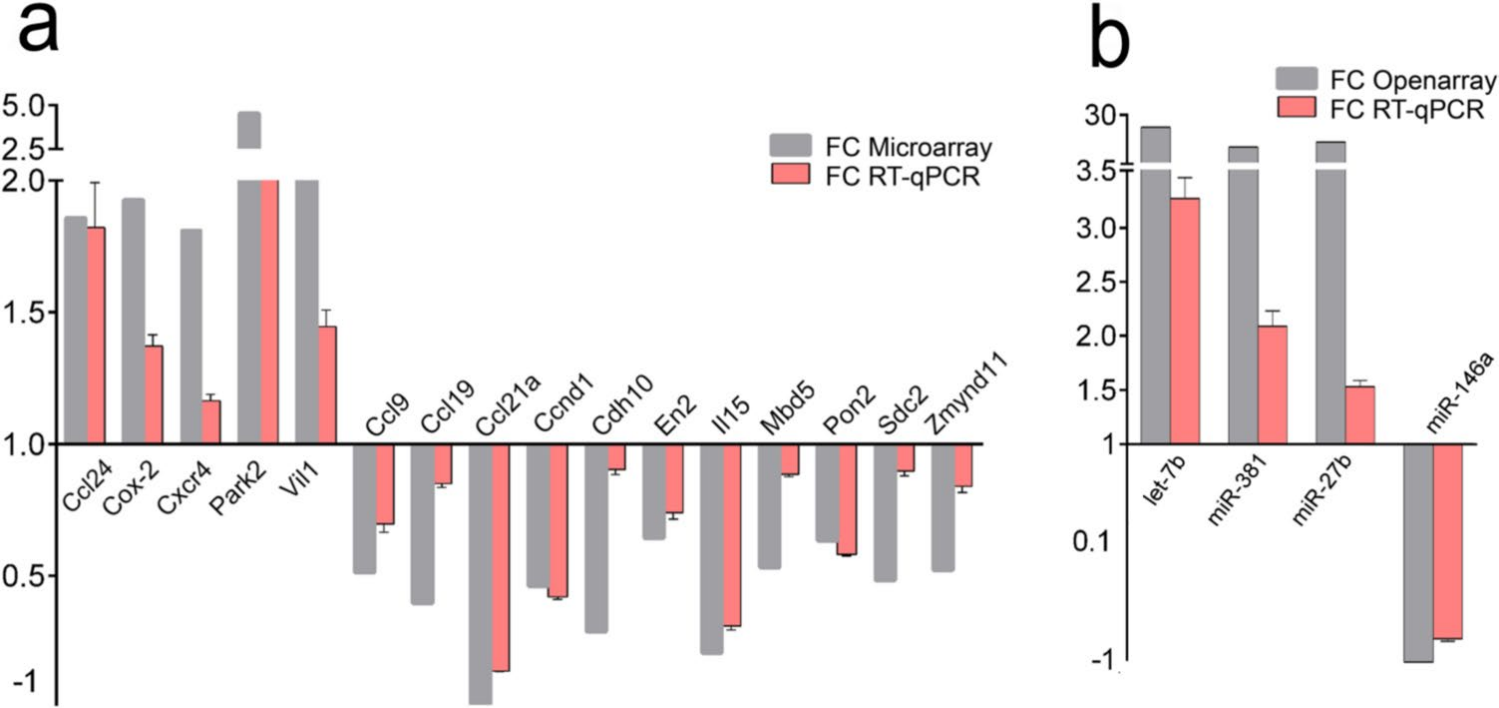
(**a**) qRT-PCR validation of DEGs. qRT-PCR results for all the evaluated genes were consistentwith microarray results. Values are expressed as comparative quantitation ratios (gene/L41), normalized to the expression levels of controls (mean ± S.E.M. of three replicates from pools of six animals per genotype; *p* < 0.05, Student’s *t*-test, BTBR vs. B6). (**b**) qRT-PCR validation of DEmiRNAs. qRT-PCR results for all the evaluated DEmiRNAs were consistent with open array results. Values are expressed as comparative quantitation ratios (miRNA/RNU19), normalized to the expression levels of controls (mean ± s.e.m. of three replicates from pools of six animals per genotype; p < 0.05, Student’s t-test, BTBR vs. B6).

We validated the microarray findings using qRT-PCR analysis, selecting sixteen representative genes from the DEG list of the BTBR PFC. These genes (*Ccl9*, *Ccl19*, *Ccl21a*, *Ccl24*, *Ccnd1*, *Cdh10*, *Cox- 2*, *Cxcr4*, *En2*, *Il15*, *Mbd5*, *Park2*, *Pon2*, *Sdc2*, *Vil1*, *Zmynd11*) were chosen based on their involvement in key pathways identified as deregulated through our enrichment analyses (DAVID, IPA, g:Profiler), including chemokine signaling, T cell receptor signaling, regulation of the actin cytoskeleton, mitochondria, inflammation, and myelination. Additionally, we selected some of these DEGs based on their presence in transcriptomic datasets from BTBR mice studies (Supplementary Table 6). Specifically, we focused on the most statistically significant dataset (i.e., Provenzano_Hippocampal_DEGs) enriched in the DEGs of the PFC in BTBR mice. To further prioritize the most relevant genes, we considered all autism-associated genes listed in the SFARI repository that were present in this dataset. From this list of 32 genes, we selected six for validation (*Cdh10*, *Cox- 2*, *Mbd5*, *Sdc2*, *Vil1*, *Zmynd11*). Furthermore, *Ccnd1*, *Cox- 2*, and *Cxcr4* were also selected based on their identification as potential targets of DEmiRNAs in our miRNA-mRNA intersection analyses. Considering the transcriptional similarities between BTBR and En2 −/− mice, we also selected the *En2* for validation [38]. All 16 genes were significantly changed in the BTBR PFC (chemokine (C–C motif) ligand 24 (*Ccl24*) *p* = 0.0128; cyclooxygenase- 2 (*Cox2*) *p* = 0.0022; C-X-C chemokine receptor type 4 (*Cxcr4*) *p* = 0.0033; Parkin (*Park2*) *p* = 0.0001; Villin 1 (*Vil1*) *p* = 0.0048; chemokine (C–C motif) ligand 9 (*Ccl9*) *p* = 0.0010; chemokine C–C motif chemokine ligand 19 (*Ccl19*) *p* = 0.0017; chemokine (C–C Motif) Ligand 21 A (*Ccl21a*) *p* = 0.0001; cyclin D1 (*Ccnd1*) *p* = 0.0001; cadherin 10 (*Cdh10*) *p* = 0.0214; engrailed 2 (*En2*) *p* = 0.0035; interleukin- 15 (*Il15*) *p* = 0.0001; methyl-CpG-binding domain protein 5, (*Mbd5*) *p* = 0.0026; paraoxonase 2 (*Pon2*) *p* = 0.0001; Syndecan- 2 (*Sdc2*) *p* = 0.0163; zinc finger MYND-type containing 1 (*Zmynd11*) *p* = 0.0138) (Fig. 9a). Furthermore, the gene expression differences reported by qRT-PCR significantly correlated with microarray data (Pearson *r* = 0.87, *p* < 0.0001).

Regarding the differentially expressed miRNAs in BTBR PFC identified through the OpenArray platform, the expression of 4 miRNAs let-7b, miR-27b, miR-146, and miR-381 was validated by qRT-PCR. Their selection was based on their roles in ASD, glial and oligodendrocyte development, and neuroinflammation. Specifically, miR-27b, miR-381, and miR-146a were linked to ASD, while miR-146a and miR-27b also demonstrated significant roles in oligodendrocyte and glial development. Additionally, let- 7b and miR- 146a were strongly associated with neuroinflammation, with let- 7b identified as a hub-miRNA in the DEmiRNA-DEG interaction network derived from IPA. Let-7b and miR-146a were also selected for validation based on the prediction that *Ccnd1* and *Cxcr4* (data not shown) are their respective gene targets, identified through the intersection analysis of DEGs and DEmiRNA target genes.

All selected miRNAs showed statistically significant differential expression in the BTBR PFC, as compared to B6 controls (miR-27b *p* = 0.0010; miR-381 *p* = 0.0021; let- 7b *p* = 0.0001; miR-146a *p* = 0.0038) (Fig. 9b). As expected, a significant correlation was observed between OpenArray and qRT-PCR results (Pearson *r* = 0.86, *p* < 0.00388).

## Discussion

This study provides a comprehensive analysis of mRNA and miRNA expression profiles in the PFC of BTBR mice, a widely used model for ASD. We found that upregulated DEGs were enriched in immune-related pathways, including chemokine signaling, T cell receptor signaling, and regulation of the actin cytoskeleton. In contrast, downregulated DEGs were associated with oligodendrocyte function, myelination, and lipid metabolism. Functional analyses of predicted target genes of downregulated DEmiRNAs were strongly associated with synaptic processes, such as synaptic signaling and transmission. Conversely, the target genes of upregulated DEmiRNAs were enriched in pathways related to Wnt signaling and insulin signaling. When analyzing correlations between DEGs and DEmiRNAs, considering their inverse relationship, common pathways emerged, including immune signaling, myelination, and neurodevelopmental processes. Through an integrated analysis, we further identified specific mRNA-miRNA-predicted interactions, uncovering extensive regulatory links and confirming a robust inverse relationship between upregulated DEGs and downregulated miRNAs, and vice versa. Notably, let- 7b emerged as the miRNA with the highest number of interactions with DEGs, suggesting its role as a potential hub miRNA. Together, the results provide evidence for miRNA control of specific dysregulated pathways in this brain region in a mouse model of ASD. The findings expand our understanding of the molecular changes in ASD and may inform the choice of potential therapeutic targets.

## Dysregulated Genes, miRNAs, Gene Ontologies, and Pathways in the PFC of BTBR Mice

Our gene expression analysis reveals that the most enriched upregulated pathways are related to inflammatory processes. Specifically, we noted a significant over-representation of chemokine, cytokine, and T cell signaling pathways, all critical for mediating inflammation. Our analysis also spotlighted a significant over-representation of cadherins, critical in cell junction formation, thereby enabling stable cell-to-cell adherence. Within the context of inflammation, cadherins might be instrumental in guiding the adhesion of immune cells to the vascular wall and their subsequent migration to inflamed tissues in ASD [65]. Cell surface interactions at the vascular wall are also highlighted as significantly enriched in the upregulated PFC DEGs of BTBR mice.

Additionally, we observed a significant overrepresentation of the calcium signaling pathway in the PFC of BTBR mice. Calcium can stimulate the production of multiple inflammatory mediators, thus amplifying the inflammatory response [66]. Another critical term upregulated is the regulation of the actin cytoskeleton, which is essential for the inflammatory response, facilitating cell movement, adhesion, shape changes, and signal transduction. While terms related to inflammatory response appear to be increased, among the significant downregulated DEGs we found GPCR (G protein-coupled receptors), in particular rhodopsin-like receptors. This group includes different types of receptors, such as neurotransmitter receptors, so their under-expression could result in impaired signal transmission and malfunction of neural networks [67]. From MPO analysis and gene set enrichment, we observed significant transcriptional changes related to glial cells, specifically astrocytes and oligodendrocytes. The upregulation of calcium signaling, a pathway correlated with astrocyte communication, along with a significant increase in astrocyte markers, suggests an activation of astrocytes in response to the neuroinflammatory process.

DEGs exhibited significant enrichment for cell-type-specific markers, with upregulated genes linked to astrocyte markers and downregulated genes showing strong enrichment for oligodendrocytes. These findings suggest distinct cell-type contributions, potentially indicating an increased number of astrocytes and a reduced number of oligodendrocytes in the BTBR prefrontal cortex. Furthermore, we observed significant enrichment in previous hippocampal and cortical transcriptome datasets of the BTBR mouse model [38, 44–46], highlighting shared transcriptional dysregulation across different brain regions in BTBR mice. Interestingly, one of these transcriptomic studies [44] also identified significantly altered pathways in line with our findings, including adherens junctions, ECM interactions, and the regulation of the actin cytoskeleton, suggesting that the altered pathways observed in the PFC may reflect a broader pattern of gene expression dysregulation in the BTBR brain and may represent potential therapeutic targets.

In addition to the specific deregulation of protein-coding genes in the PFC of BTBR mice, the present study also investigated alterations in miRNA expression. This builds on previous work that identified changes in miRNA expression associated with ASD in both human samples and animal models [58]. Initially, we investigated the potential biological roles of each DEmiRNA through a literature-based analysis, which highlighted several key miRNAs, including miR-27b, miR-146a, miR-23a, and miR-328. These miRNAs are implicated in ASD, oligodendrocyte and glial development, and neuroinflammation, emphasizing their multifaceted biological roles and potential as therapeutic targets. In the context of oligodendrocyte and glia development, miR- 23a, miR- 338, miR- 146a, and miR- 27b were shown to have pivotal roles in lineage progression, particularly by regulating differentiation processes and myelin sheath formation [68–72]. Notably, miR- 106b and miR- 199a emerged as regulators of neural stem/progenitor cell proliferation, neuronal differentiation, and oligodendrocyte maturation, further underscoring their importance in neural and glial cell biology [73, 74].

Regarding neuroinflammation, miR-146a stands out as a key regulator of inflammatory responses via the NF-κB and IRAK1 pathways, contributing to cellular differentiation and immune modulation [61]. Similarly, miR-23a and miR-338 have been implicated in the regulation of metabolic pathways and inflammatory signaling, while miR- 592 and let- 7b have been linked to oxidative stress and microglial activation, respectively [60, 61]. Additionally, let- 7b has been highlighted for its role in TLR7 activation, further underscoring its involvement in neuroinflammatory pathways [60].

Gene targets of DEmiRNAs showed significant enrichment in ASD- and schizophrenia-related genes derived from a large-scale RNA-Seq analysis of the cerebral cortex [43], suggesting that conserved genetic mechanisms may underlie shared co-morbidities observed in these two neurodevelopmental disorders and highlighting conserved molecular signatures between BTBR mice and human conditions, which reinforce the translational relevance of this mouse model for studying the underlying mechanisms of ASD.

Notably, neither the DEGs nor the predicted targets of the DEmiRNAs displayed significant enrichment for autism-related genes, such as those compiled within the SFARI repository. These findings align with a recent study indicating that, although SFARI genes are regarded as strong ASD candidates, they exhibit only minimal differential expression in ASD [75]. Although SFARI genes generally display higher baseline expression than other neuronal genes, their overall shifts in ASD contexts remain modest, with smaller fold changes and subtle expression differences [75]. Moreover, SFARI genes are implicated in a range of neurodevelopmental disorders including schizophrenia and bipolar disorder, indicating broader functional relevance. This could explain the lack of SFARI gene enrichment in BTBR mice, suggesting that ASD-like phenotypes in this model may arise through alternative or complementary pathways, rather than being driven by expression changes in SFARI genes.

Interestingly, the pathway analysis highlighted that the most enriched pathways are associated with the predicted target genes of downregulated miRNAs, primarily involving inflammation and the immune system such as chemokine, T cell signaling pathways, and leukocyte migration. Our findings also show that several terms associated with synaptic signaling and plasticity are affected by downregulated miRNAs, suggesting compromised functionality in these processes. On the other hand, pathways related to target genes of upregulated miRNAs include insulin and Wnt signaling pathways, which may influence brain circuits involved in mood and cognition and contribute to brain development and function, potentially leading to the onset of ASD, as observed in other mouse model studies on the PFC [76, 77].

Furthermore, our analysis indicated that some of the pathways related to target genes of upregulated miRNAs consistently share similar dysregulation within the downregulated DEGs in BTBR mice. Our results suggest shared features, particularly in relation to the myelination process and neuroglial cells. The reduced expression of genes involved in the processes of genesis, development, and differentiation of glial cells could reflect incomplete maturation of the central nervous system, implying a slowing of the conduction of nerve impulses. The dysregulation observed in pathways related to myelination and oligodendrocyte functions aligns with findings from other studies, including the analysis of mRNA and miRNA expression profiles in the cerebral cortex of the BTBR mouse model [46]. Similar patterns have also been observed in cortical transcriptomic profiles of other ASD mouse models [78]. Although our analysis specifically focused on the PFC, it underscores the importance and generalizability of myelination dysfunctions across cortical regions in ASD. Furthermore, hypomyelination in the medial prefrontal cortex of BTBR mice, indicative of altered neurodevelopmental trajectories, has been also demonstrated [79].

Other interesting pathways identified in both downregulated DEGs and upregulated miRNA target genes in our study include dendritic branching, mitochondria, energy expenditure, and the ECM. Dysfunctions in these pathways could critically impact synaptic connectivity, neural signal transmission, brain plasticity, and metabolic processes essential for sustaining neuronal activity. Similarly, shared features were consistently found between target genes of downregulated miRNA and upregulated DEGs. Here, all pathway analyses support a relevant role in cell migration and localization, especially in leukocyte movement. The presence of terms related to an increased quantity of T lymphocytes and activation of the chemokine signaling pathway suggests an active inflammatory response in the PFC of BTBR mice. Finally, we identified several terms associated with the development of the nervous and sensory systems, as well as head morphology. Alterations in these genes may relate to morphological anomalies in BTBR mice, such as reduced PFC size [27, 35].

Our analysis also revealed distinct functional effects of DEmiRNAs compared to DEGs, including the enrichment of synaptic genes among the predicted targets of DEmiRNAs, a process not observed when analyzing DEGs alone. This finding emphasizes the complexity of post-transcriptional regulation and could indicate processes that evade miRNA control in BTBR mice. However, while miRNA profiling alone may not fully capture all downstream effects on target genes, mRNA profiling by itself cannot account for the complete spectrum of miRNA-mediated regulation. These results highlight the importance of integrating miRNA and mRNA analyses to achieve a more comprehensive understanding of regulatory dynamics. Furthermore, incorporating other data types, such as proteomics, could provide deeper insights into the diverse actions of miRNAs and their downstream effects.

We performed an mRNA-miRNA pair interaction analysis to investigate the complex relationships between DEGs and DEmiRNAs. While previous analyses highlighted the convergent pathways between DEGs and DEmiRNAs, this analysis explored their multiple interactions within interconnected pathways. Notably, miRNAs such as let-7b, miR-143, and miR-34b emerged as central regulatory hubs, interacting extensively with several genes critical to neurogenesis, inflammation, and cell cycle regulation, such as *Tp53*, *E2f1*, *E2f2*, *Npm1*, *Rela*, *Relb*, *Pparg*, and *Bcl6* [46, 80–82]. Of particular interest was the identification of over-expressed let- 7b, which exhibited the highest number of interactions with downregulated DEGs, highlighting its crucial regulatory role and underscoring its potential as a therapeutic target.

All sixteen selected genes validated by RT-qPCR analysis showed a significant change in expression. These genes were identified based on their involvement in key deregulated pathways and their relevance in previous studies of BTBR mice and autism-related datasets. This included upregulation of inflammation-related *Ccl24* and *Cxcr4*. Elevated levels of *Ccl24* have been consistently detected in the serum and cerebrospinal fluid (CSF) across various neurodegenerative diseases [83]. Meanwhile, *Cxcr4*, a pivotal chemokine receptor, plays a critical role in orchestrating diverse functions within the immune system and neurodevelopmental processes [84]. In contrast, abnormalities in the expression of *Ccnd1*, which has been linked to schizophrenia and to reduction in brain oligodendrocytes [85], along with *Pon2*, uniquely expressed in brain tissue among the PON family enzymes and renowned for its antioxidant and anti-inflammatory properties [86], were found to be downregulated.

Among other validated genes, *Cadherin- 10 (Cdh10)*, *En2*, *Mbd5*, and *Syndecan- 2 (Sdc2)*, known for their roles in synaptic function, neurodevelopment, and ASD, were significantly downregulated *Cdh10* is crucial for maintaining the balance between excitatory and inhibitory synaptic functions in cortical neurons [87]. *En2* is associated with ASD in several studies [88, 89], and mice lacking the gene exhibit significant transcriptional similarities to the hippocampus of the BTBR mouse [38]. *Mbd5* haploinsufficiency has been associated with epilepsy, intellectual disability, and ASD [90–92] and its deficiency in mice leads to abnormal social behavior and impaired learning [93]. *Sdc2* is critical for synaptic organization where the protein product interacts with ECM components and plasma membrane molecules [94]. Conversely, *Vil1*, which is upregulated, encodes a calcium-regulated actin-binding protein, potentially influencing calcium signaling and actin-regulated cell movement [95]. *Cox- 2*, also upregulated, has been identified as a possible target to reduce inflammation in neuropsychiatric disorders, including ASD [96], suggesting its relevance for therapeutic exploration in the BTBR mouse model. Four selected miRNAs were validated by RT-qPCR (miRNA- 27b, miRNA-let- 7b, miR-146a, miR-381). These have been linked to neuropsychiatric diseases, glial and oligodendrocyte development, and inflammation. Increased miRNA- 27b expression has been linked to neuroinflammatory states and the pathological differentiation of T cells in multiple sclerosis [97], possibly hinting at similar mechanisms in the BTBR model. Let- 7b, which we identified as a hub-miRNA in the DEmiRNA-DEGs interaction network derived from IPA, is associated with innate immunity activation and neurodegeneration [98]. Let- 7b has also been linked to cognitive impairments in autism and depression [99, 100].

MiR-146a is known for promoting oligodendrocyte genesis and reducing inflammation, with its loss affecting the differentiation of stem cells and glial cells, and which is associated with developmental brain disorders and abnormal synaptic maturation [69, 101–103]. Finally, our finding of the overexpression of miR-381 aligns with findings from other studies on cortical samples from post-mortem ASD patients [58, 104].

There are a number of limitations of the present study. The use of the B6 strain as a control presents certain limitations. B6 and BTBR belong to distinct branches of the mouse genetic tree, raising concerns about its suitability as an ideal reference strain for transcriptomic profiling [105]. Lacking a more suitable control strain, we conformed to previous studies using B6 as a control for transcriptional profiling analyses [106]. Future studies should prioritize identifying or developing more genetically appropriate control strains to reduce potential biases related to genetic divergence. Not all mRNA changes could be directly linked to alterations in miRNA expression, underscoring the complexity of gene regulation in neurodevelopmental disorders and implying the involvement of other regulatory mechanisms, such as transcription factors or chromatin modifications. Future studies should aim to integrate protein data to more completely assess any miRNA-dependent actions on pathways, and additional layers of regulation, including long non-coding RNAs and epigenetic modifications, to provide a more comprehensive understanding of the regulatory networks in ASD. The complexity of the human prefrontal cortex presents challenges in fully replicating its features in a mouse model like the BTBR. Similarly, the behavioral phenotypes observed in BTBR mice, while relevant to core ASD features, may not encompass the full spectrum of ASD-associated behaviors or comorbidities. However, this model offers valuable insights into PFC-related neural mechanisms and can enhance our understanding of ASD.

While array-based methods have limitations in covering the entire transcriptome, RNA sequencing offers a more comprehensive and unbiased view of gene expression. Moving forward, integrating mRNA and miRNA analysis to determine if specific biological processes are cell type-specific could significantly advance our understanding of complex biological disorders such as ASD.

Lastly, our study focused on the PFC, a region implicated in ASD pathophysiology. However, ASD is a multisystem disorder involving multiple brain regions and systems. Future research should expand the analysis to other regions, such as the hippocampus and cerebellum, to capture a more holistic understanding of the molecular and functional dysfunctions in ASD.

## Conclusions

Although there is currently no specific diagnostic biomarker or treatment for autism, advances in genetics have identified numerous genes associated with the disorder, which provide molecular targets for both mechanistic insights and potential interventions. Transcriptomic analyses in individuals with autism have highlighted widespread changes in gene expression, particularly in pathways related to cell stress and neuroinflammation. These are thought to play a critical role in the behavioral and cognitive impairments observed in ASD [3, 4, 6, 107]. Building on these findings, our study on the PFC of BTBR mice identifies gene expression changes and miRNA dynamics that could illuminate the complex biological processes mediating the ASD-like behaviors observed in these mice.

Our analysis showed that most deregulated gene pathways coincided with predicted mRNA targets of miRNAs, indicating almost complete convergence and suggesting significant miRNA influence. This inverse relationship implies a regulatory mechanism in which decreased miRNA expression may lead to increased expression of target mRNAs, thereby enhancing activity in these pathways commonly implicated in ASD pathophysiology.

Our findings emphasize the importance of integrating miRNA and mRNA profiles to unravel the molecular processes affecting the normal functioning of the PFC, which in turn impacts cognitive, social, and executive functions. They also illuminate the critical interplay between inflammation, neuroglial modulation, myelination, extracellular matrix interactions, synaptic functions, and cell–cell communication. These results represent a step toward developing targeted interventions based on miRNA levels and their potential mRNA targets, providing a promising path for addressing specific behavioral deficits in BTBR mice with potential implications for preclinical and clinical studies.

## Supplementary Information

Below is the link to the electronic supplementary material.

ESM 1 (XLSX 373 KB)

ESM 2 (XLSX 48.6 KB)

ESM 3 (XLSX 146 KB)

ESM 4 (XLSX 248 KB)

ESM 5 (XLSX 31.1 KB)

ESM 6 (XLSX 27.7 KB)

ESM 7 (XLSX 1.52 MB)

ESM 8 (XLSX 22.6 KB)

ESM 9 (XLSX 30.4 KB)

ESM 10 (XLSX 96.7 KB)

ESM 11 (XLSX 25.4 KB)

ESM 12 (XLSX 28.9 KB)

ESM 13 (XLSX 104 KB)

ESM 14 (XLSX 29.5 KB)

ESM 15 (DOCX 306 KB)

## Data Availability

PFC gene expression datasets of BTBR and B6 mice have been deposited in GEO under accession numbers GSE81502 (mRNA) and GSE87601 (miRNA).

## Materials Availability

All data needed to evaluate the conclusions in the paper are present in the paper.
