# Supplementary material for: Deregulated mRNA and microRNA Expression Patterns in the Prefrontal Cortex of the BTBR Mouse Model of Autism
Source: Mol Neurobiol. 2025 Apr 14;62(8):10614–34. doi: 10.1007/s12035-025-04900-x (PMC12289737; doi:10.1007/s12035-025-04900-x)
Supplement: Supplementary file 15 — (DOCX 306 KB) [file 12035_2025_4900_MOESM15_ESM.docx]

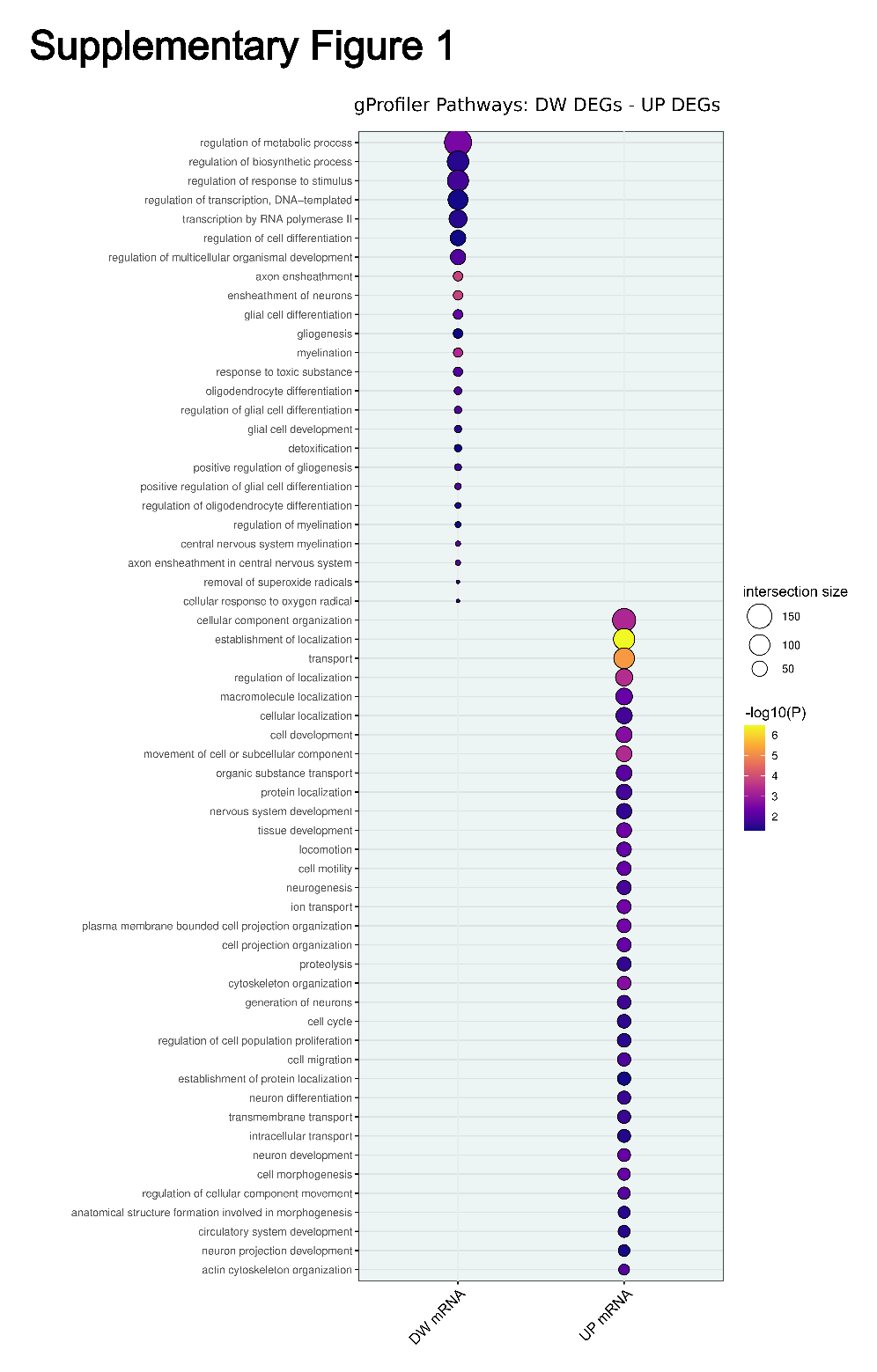


**Supplementary Figure 1:** **g:Profiler functional analysis for down- and up-regulated DEGs.**

The plot illustrates the Gene Ontology terms with the most significant adjusted p-values, excluding redundant or overly generic terms. Only terms with an adjusted p-value (<0.05) were considered. For each biological term reported on the y-axis, balloon size is proportional to the number of genes involved in the category, while color represents the negative logarithm of the corrected p-value.
